# Association between Hepatitis C Virus Infection and Esophageal Cancer: An Asian Nationwide Population-Based Cohort Study

**DOI:** 10.3390/jcm10112395

**Published:** 2021-05-28

**Authors:** Yin-Yi Chu, Jur-Shan Cheng, Ting-Shu Wu, Chun-Wei Chen, Ming-Yu Chang, Hsin-Ping Ku, Rong-Nan Chien, Ming-Ling Chang

**Affiliations:** 1 Division of Gastroenterology, Department of Gastroenterology and Hepatology, Chang Gung Memorial Hospital, Taoyuan 333423, Taiwan; chu2235@yahoo.com (Y.-Y.C.); 8902088@cgmh.org.tw (C.-W.C.); 2 Department of Gastroenterology and Hepatology, New Taipei Municipal Tu Cheng Hospital, New Taipei City 236, Taiwan; find94132@yahoo.com; 3 Department of Medicine, College of Medicine, Chang Gung University, Taoyuan 333323, Taiwan; tingshu.wu@gmail.com (T.-S.W.); p123073@gmail.com (M.-Y.C.); ronald@adm.cgmh.org.tw (R.-N.C.); 4 Clinical Informatics and Medical Statistics Research Center, College of Medicine, Chang Gung University, Taoyuan 333423, Taiwan; jscheng@mail.cgu.edu.tw; 5 Department of Emergency Medicine, Chang Gung Memorial Hospital, Keelung 20401, Taiwan; 6 Division of Infectious Diseases, Department of Internal Medicine, Linkou 333423, Taiwan; 7 Division of Pediatric Neurologic Medicine, Chang Gung Children’s Hospital, Taoyuan 333423, Taiwan; 8 Division of Pediatrics, Chang Gung Memorial Hospital, Keelung 20401, Taiwan; 9 Division of Hepatology, Department of Gastroenterology and Hepatology, Chang Gung Memorial Hospital, Taoyuan 333423, Taiwan

**Keywords:** HCV, esophageal cancer, male, interferon, mortality

## Abstract

**Background:** Hepatitis C virus (HCV) infection causes many extrahepatic cancers, and whether HCV infection is associated with esophageal cancer development remains inconclusive. **Methods:** A nationwide population-based cohort study of the Taiwan National Health Insurance Research Database (TNHIRD) was conducted. **Results:** From 2003 to 2012, of 11,895,993 patients, three 1:1:1 propensity score-matched cohorts, including HCV-treated (interferon-based therapy ≧6 months, *n* = 9047), HCV-untreated (*n* = 9047), and HCV-uninfected cohorts (*n* = 9047), were enrolled. The HCV-untreated cohort had the highest 9-year cumulative incidence of esophageal cancer among the three cohorts (0.174%; 95% confidence interval (CI): 0.068–0.395) (*p* = 0.0292). However, no difference in cumulative incidences was identified between the HCV-treated (0.019%; 0.002–0.109%) and HCV-uninfected cohorts (0.035%; 0.007–0.133%) (*p* = 0.5964). The multivariate analysis showed that HCV positivity (hazard ratio (HR): 5.1, 95% CI HR: 1.39–18.51) and male sex (HR: 8.897; 95% CI HR: 1.194–66.323) were independently associated with the development of esophageal cancer. Of the three cohorts, the HCV-untreated cohort had the highest cumulative incidence of overall mortality at 9 years (21.459%, 95% CI: 18.599–24.460) (*p* < 0.0001), and the HCV-treated (12.422%, 95% CI: 8.653–16.905%) and HCV-uninfected cohorts (5.545%, 95% CI: 4.225–7.108%) yielded indifferent cumulative mortality incidences (*p* = 0.1234). **Conclusions:** Although HCV positivity and male sex were independent factors associated with esophageal cancer development, whether HCV infection is the true culprit or a bystander for developing esophageal cancer remains to be further investigated. Interferon-based anti-HCV therapy might attenuate esophageal risk and decrease overall mortality in HCV-infected patients.

## 1. Introduction

Esophageal cancer is the sixth leading cause of cancer death in males, with an estimated >500,000 new cases and >500,000 deaths annually, accounting for 3.2% of cancer cases and 5.37% of cancer deaths worldwide [1]. China, South Africa, and North Central Asia are considered to have the highest incidence of esophageal cancer [2]. The 5-year survival rate among patients with esophageal cancer is 19%, decreasing to 0.9% in patients with advanced esophageal cancer [3]. There are two main histological types of esophageal cancer: esophageal squamous cell carcinoma (ESCC) and esophageal adenocarcinoma (EAC). The risk factors for esophageal SCC include alcohol, smoking, betel nut chewing [4], and hypertension [5]. Metabolic syndrome (MetS) [6] and body mass index (BMI)/obesity [5,7] are related to a higher risk of EAC. In contrast, a high BMI significantly decreases the risk of ESCC [5].

Hepatitis C virus (HCV) is a human pathogen responsible for acute and chronic liver disease that infects an estimated 150 million individuals worldwide [8]. In addition to hepatic complications such as steatosis, cirrhosis, and hepatocellular carcinoma (HCC), HCV causes extrahepatic complications, including mixed cryoglobulinemia [9], dyslipidemia, diabetes [10], obesity, cardiovascular events [11], and neurological manifestations [12]. Moreover, HCV infection is associated with many extrahepatic malignancies, including lymphoid [13], head and heck [14], thyroid [15], lung, pancreas, kidney [13], and gastric cancers [16], B-cell non-Hodgkin’s lymphomas, and intrahepatic cholangiocarcinoma [17]. However, the link between HCV infection and esophageal cancer has been elucidated but remains inconclusive. An Asian study showed that compared with HCV seronegative patients, HCV seropositive patients had a higher multivariate-adjusted hazard ratio (HR) for esophageal cancer [15], while another study of a U.S. population showed that esophageal cancers were not more frequent among HCV-infected patients compared with the general population [13]. Combination therapy with pegylated interferon (Peg-IFN) and ribavirin has provided a “cure” for a considerable proportion of HCV-infected patients, particularly in those with the favorable interferon λ 3 (IFNL3) genotype [8]. The cure rates were further improved by replacing interferon-based therapy with direct-acting antiviral agents (DAAs) [8], which led to a sustained virological response (SVR) rate as high as 100% [18]. However, HCV-associated malignancies are not eradicable, especially among patients with baseline diabetes and cirrhosis [19,20]. Whether HCV infection accelerates the risk of esophageal cancer is still a crucial issue in the era of using DAA to eliminate HCV infection.

Accordingly, we aimed to examine the impacts of HCV infection on the development of esophageal cancer in Taiwan, an Asian country where HCV infection is rampant [21], by conducting a nationwide population-based cohort study using the Taiwan National Health Insurance Research Database (TNHIRD). The cumulative incidences of esophageal cancer and esophageal cancer-associated mortalities among HCV-infected subjects with and without anti-HCV therapy and HCV-uninfected subjects were compared to explore the impacts of HCV infection and anti-HCV therapy.

## 2. Methods

### 2.1. Samples and Measurements

National-level data, including the National Health Insurance (NHI) administrative database, the Cancer Registry Database, and the Death Registry Database, were used to retrieve data for this population-based retrospective cohort study. The NHI program is a mandatory, single-payer system that covers >99% of the population and offers comprehensive coverage, ranging from laboratory tests and prescription drugs to ambulatory care and hospital services. The HCV-treated cohort consisted of patients who had an HCV RNA test and received Peg-IFN and ribavirin (RBV) for more than 6 months between 1 January 2003 and 31 December 2012. The date of their first HCV test was the index date. The baseline was defined as the date of six months after completing the combination therapy, which was the time to ensure SVR. Patients with cirrhosis-related complications, including hepatoencephalopathy, esophageal or gastric varices, ascites or hepatorenal syndrome, were excluded to avoid interference from these complications or from the associated treatment on the development of esophageal cancer. Those diagnosed with esophageal cancer and those who died before baseline were also excluded. The HCV-untreated subjects included patients who met all the following criteria: (1) received HCV tests (HCV antibody or HCV RNA test); (2) had a diagnosis of HCV infection (International Classification of Diseases, Ninth Revision, Clinical Modification (ICD-9-CM) codes: 070.41, 070.44, 070.51, 070.54, 070.70, 070.71, V02.62)); (3) received hepatoprotective agent therapy, including silymarin, liver hydrolysate, choline bitartrate, and ursodeoxycholic acid; and (4) did not have any history of anti-HCV therapy (Peg-IFN or RBV). Their index date was the date of their first HCV test. The HCV-uninfected individuals consisted of those without any HCV diagnosis, HCV tests, and hepatoprotective agent therapy or anti-HCV treatment. Their index date was the date of one of their physician visits randomly selected from their claims database. The HCV-treated cohort was 1:1:1 matched with the HCV-untreated cohort and with the HCV-uninfected cohort using a propensity score-matched method to assure comparable observed characteristics among the three cohorts. The probability of receiving the combination therapy was estimated by adopting a logistic model with the following covariates: age, NHI registration location, Charlson Comorbidity Index (CCI) score [22], and year of the index date. In the HCV-untreated and HCV-uninfected cohorts, the baseline was determined based on the time elapsed from the index date to the baseline of their matched HCV-treated counterparts. The same exclusion criteria were applied to the HCV-untreated and HCV-uninfected cohorts. The matching processes of the three cohorts are demonstrated in Supplementary Figure S1.

Development of esophageal cancer (ICD-9-CM:150) was identified from the Cancer Registry data that provides types of cancers and dates of diagnosis. Esophageal cancer-related mortality (ICD-9-CM code: C15) was retrieved from the Death Registry data that contains information on causes and dates of death. Subjects were followed from baseline until the date of event (esophageal cancer or esophageal cancer-related mortality), death, or the end of follow-up (31 December 2013), whichever occurred first.

### 2.2. Statistical Analysis

Statistical Package for Statistical Analysis System (SAS version 9.4, SAS Institute Inc., Cary, NC, USA) software was used to perform the analyses. The modified Kaplan–Meier method and the Gray method that took into account death as a competing risk event [23] were used to estimate and compare cumulative incidences. A subdistribution hazards model [24], which is a modified Cox proportional hazards model that considers competing mortality, was used to estimate the adjusted HR of esophageal cancer development. Covariates of the models included age, sex, NHI registration location, CCI score, year of the index date, baseline liver cirrhosis, end-stage renal disease (ESRD), chronic obstructive pulmonary disease (COPD), diabetes mellitus (DM), hypertension, dyslipidemia, cardiovascular events, and stroke. Cardiovascular events consisted of percutaneous coronary intervention, myocardial infarction, cardiogenic heart failure, shock, coronary artery bypass graft, and peripheral vascular disease. Statistical significance level was defined at 5%.

### 2.3. Informed Consent

The study protocol conformed to the ethical guidelines of the 1975 Declaration of Helsinki and was approved by the Chang Gung Medical Foundation Institutional Review Board. The need for consent was waived because the national-level data used in this study were deidentified by encrypting personal identification information.

## 3. Results

### 3.1. Baseline Characteristics

**Figure 1:**
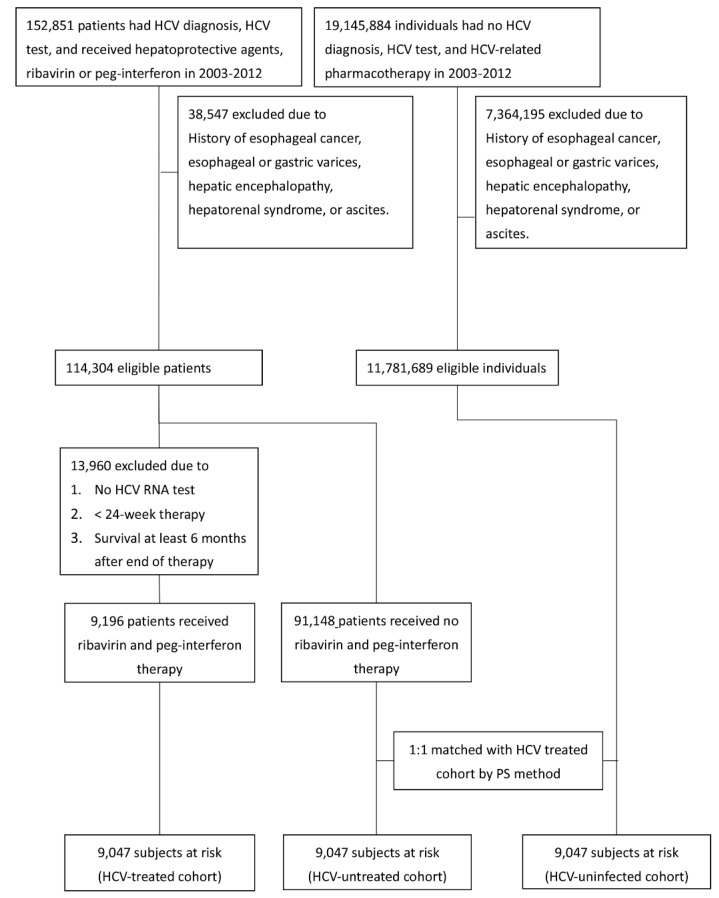
Flow chart of TNHIRD study subjects’ selection. TNHIRD: Taiwan National Health Insurance Research Database; HCV: hepatitis C virus; Peg-IFN: pegylated interferon; PS: propensity score.

From a total of 19,298,735 individuals assessed between 1 January 2003 and 31 December 2012, 11,895,993 patients without baseline esophageal cancer were identified; 114,304 patients with HCV infection and 11,781,689 patients without HCV infection were eligible for the study. In total, three cohorts, including HCV-treated (*n* = 9047), HCV-untreated (*n* = 9047) and HCV-uninfected (*n* = 9047) cohorts, were enrolled (Figure 1). The three cohorts were matched with the propensity scores and did not differ in demographic factors, residency, CCI score or index year, which were the covariates in the models to calculate propensity scores, although baseline comorbidities were not similar (Table 1). Compared with HCV-untreated cohorts, the HCV-treated cohort had higher rates of baseline cirrhosis but comparable rates of COPD and lower rates of other comorbidities. Compared with the HCV-uninfected cohort, the HCV-treated cohort had higher rates of baseline cirrhosis, COPD, ESRD, and hypertension but lower rates of dyslipidemia and stroke. Compared with the HCV-uninfected cohort, the HCV-untreated cohort had higher rates of all baseline comorbidities except dyslipidemia and stroke. To determine the HCV-associated complications, we compared the baseline factors between the HCV-infected cohort (which was a combination of the HCV-treated and HCV-untreated cohorts) and HCV-uninfected cohort. The HCV-infected cohort had higher rates of all baseline comorbidities except dyslipidemia and stroke for which there were lower rates than that of the HCV-uninfected cohort (Supplementary Table S1).

### 3.2. Cumulative Incidences and Associated Factors of Esophageal Cancer

**Figure 2:**
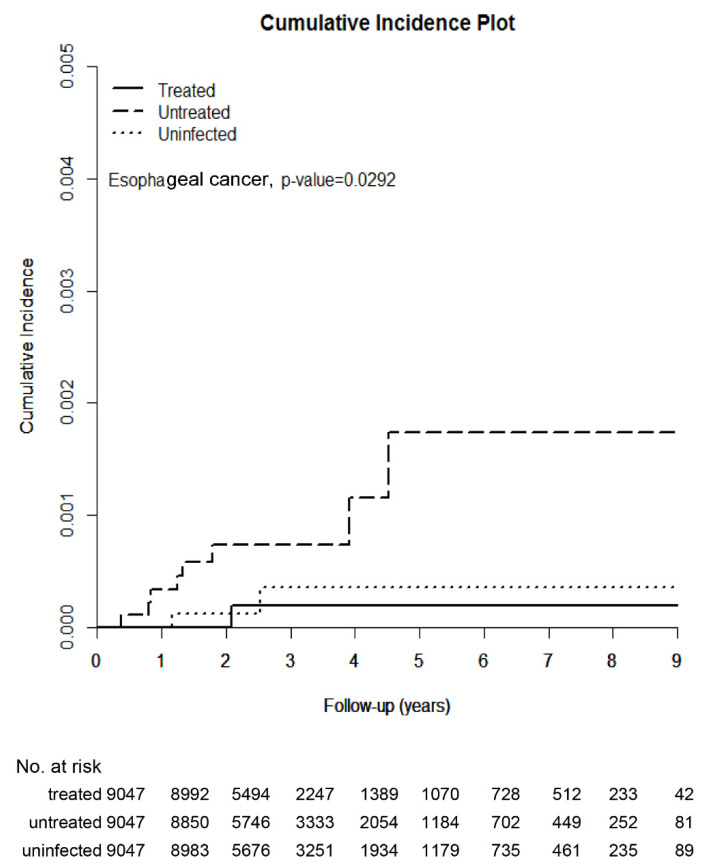
Cumulative incidence of esophageal cancers among the three TNHIRD cohorts, including HCV-treated, HCV-untreated, and HCV-uninfected cohorts.

**Figure 3:**
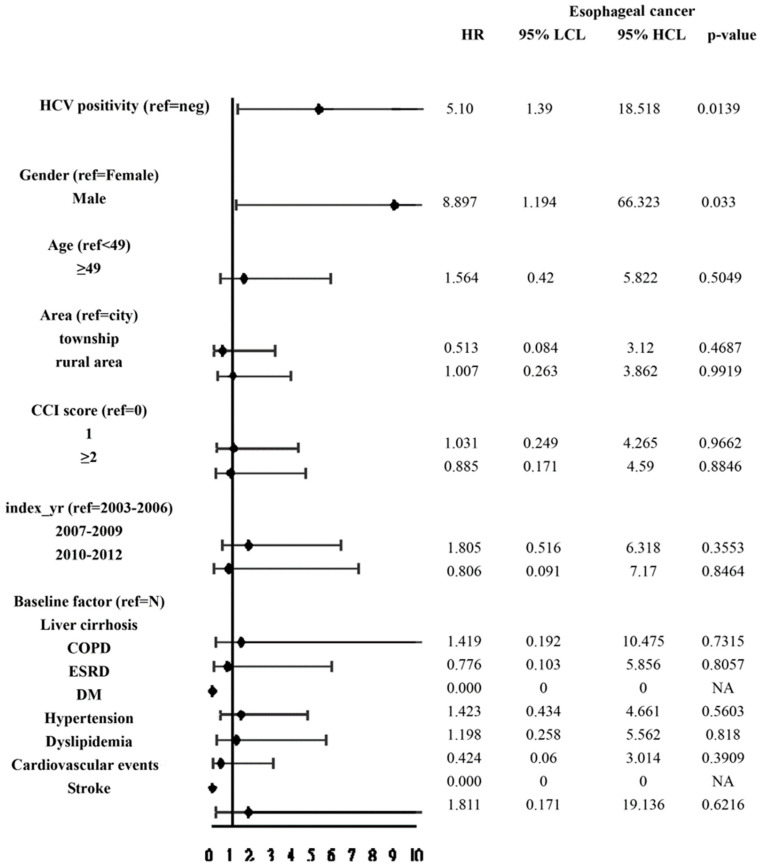
Forest plot of factors associated with incident esophageal cancers in the two TNHIRD cohorts, including HCV-positive and HCV-negative cohorts. neg: Negative; HR: hazards ratio; CI: confidence interval; LCL: lower confidence limit; HCL: higher confidence limit; HCV: hepatitis C virus; COPD: Chronic obstructive pulmonary disease; ESRD:end stage renal disease; DM: diabetes.

The HCV-treated, untreated, and uninfected cohorts were followed up until death for a duration of up to 9 years. The HCV-untreated cohort had the highest cumulative incidence of esophageal cancer among the three cohorts (Figure 2, Table 2). However, no difference in cumulative incidences of esophageal cancer was identified between the HCV-treated and HCV-uninfected cohorts (*p* = 0.5965). The multivariate analysis of the three cohorts showed that male patients had a higher hazard ratio (HR: 8.894, 95% confidence interval (CI) of HR: 1.194–66.227) than female patients; compared with the HCV-untreated cohort, the HCV-treated cohort had a borderline lower HR (*p* = 0.054) (Supplementary Figure S2). Because the HCV-treated and HCV-uninfected cohorts yielded similar cumulative incidences of esophageal cancer, we combined the HCV-treated and HCV-uninfected cohorts to form an HCV-negative cohort to determine the impact of the presence of HCV on the development of esophageal cancer. Compared with the HCV-negative cohort, the HCV-positive (i.e., HCV-untreated) cohort had a higher risk of incident esophageal cancer (*p* < 0.0001). The multivariate analyses of these two cohorts showed that HCV positivity (HR: 5.1, 95% CI HR: 1.39–18.51) and male sex were independently associated with the development of esophageal cancer (Figure 3).

### 3.3. Mortality

Of the three cohorts, the HCV-untreated cohort had the highest cumulative incidence of overall mortality at 9 years (21.459%, 95% CI: 18.599–24.460) (*p* < 0.0001), and the HCV-treated (12.422%, 95% CI: 8.653–16.905%) and HCV-uninfected (5.545%, 95% CI: 4.225–7.108%) cohorts yielded nonsignificant mortality rates (*p* = 0.1234). No differences in esophageal cancer-associated mortality were noted among the three cohorts (*p* = 0.1966).

## 4. Discussion

The most compelling results of the current study were as follows: (1) The HCV-untreated cohort had the highest 9-year cumulative incidence of esophageal cancer among the three cohorts, while no difference in cumulative incidences was identified between the HCV-treated and HCV-uninfected cohorts; (2) HCV positivity and male sex were independent factors associated with esophageal cancer development; and (3) The HCV-untreated cohort had the highest cumulative incidence of overall mortality, while the HCV-treated and HCV-uninfected cohorts yielded nonsignificant mortality incidences.

At baseline, the findings that the HCV-treated cohort had a higher cirrhosis rate than the untreated cohort and that the HCV-infected cohort had higher cirrhosis and cardiometabolic complication rates but lower dyslipidemia rates than the HCV-uninfected cohorts were consistent with the ideas that only patients with significant fibrosis were reimbursed for interferon-based anti-HCV therapy by NHI, Taiwan [25], and HCV infection causes cirrhosis, cardiometabolic events, and hypolipidemia [8]. These different baseline comorbidities thus supported the reliability of the results based on TNHIRD.

The fact that the HCV-untreated cohort had the highest esophageal cancer cumulative incidence, and that HCV positivity was independently associated with esophageal cancer development suggests that HCV infection might increase the risk of esophageal cancer, although the reported data are conflicting, as mentioned above [13,15], and different ethnicities might account for the discrepancy. More than 90% of patients with esophageal cancer in Asian countries suffer from ESCC [26]. In contrast, in Western countries, most esophageal cancers are EACs [5]. Interestingly, both current and other cohort studies observing the positive link between HCV infection and esophageal cancer were conducted in Taiwan, an Asian country endemic for the practice of Areca nut and betel quid chewing [27], and a negligible association between HCV infection and esophageal cancers was noted in the U.S. population [13]. In particular, in Taiwan, there is an increasing trend of the incidence of ESCC but not that of EAC [26]. Moreover, betel nut chewing has been regarded as a risk factor for HCV infection in Taiwan [28]. Although MetS was associated with poor prognosis in ESCC patients [29], as mentioned, MetS, including increased BMI, decreases the risks of ESCC [5]. Together, the connection between HCV infection and esophageal cancer might accelerate ESCC through a betel liquid chewing habit in HCV-infected patients rather than through metabolic alteration subsequent to HCV infection to accelerate EAC, based on the unique pathology trend of esophageal cancer and the betel liquid chewing practices of Taiwan [27]. Of note, the fact that the cumulative incidences of esophageal cancer were similar between the HCV-treated and HCV-uninfected cohorts suggests that the HCV-associated esophageal cancer risk might be reversed by interferon-based anti-HCV therapy. Future studies are needed to verify the reversibility of esophageal cancer risk in HCV-infected patients with viral clearance following DAA therapy [8].

In addition to HCV positivity, male sex was independently associated with the cumulative incidence of esophageal cancer among the three TNHIRD cohorts. Consistently, the risks of developing esophageal cancer among men have increased worldwide [30]. It is possible that the risk factors for the development of ESCC [4,5] are more common among men, which potentially, at least partly, explains the higher cumulative incidence of esophageal cancer among men. Thus, male HCV-infected patients have a higher risk of esophageal cancer, regardless of anti-HCV therapy.

Although the esophageal cancer-associated mortality was similar among the three cohorts, the HCV-untreated cohort yielded the highest overall mortality, which might be caused by HCV-associated events, such as cirrhosis, HCC or cardiometabolic events [8] other than esophageal cancer-associated complications, as no difference in esophageal cancer-associated mortality was noted among the three cohorts. This phenomenon indicates the importance of prescribing anti-HCV therapy in HCV-infected patients to decrease overall mortality.

There are limitations in the current study. First, because linking the results from TNHIRD to the laboratory results of individual patients was forbidden, the correlation of SVR with esophageal cancer could not be identified. Regardless, we are confident of the antiviral efficacy in the HCV-treated cohort because interferon-based therapy for HCV infection generally achieves an SVR rate ranging from 70% to 90% in Taiwan [31], where favorable genetic variation in IFNL3is prevalent [32]. Second, HCV testing is not universally performed in Taiwan, and HCV-uninfected individuals were the patients who did not receive any HCV diagnosis, HCV tests, hepatoprotective agent therapy, or anti-HCV treatment. There might be undiagnosed HCV-infected patients in the HCV-uninfected cohort. However, because the reimbursement of anti-HCV therapy in Taiwan is nationwide and only up to 2.7% of the Taiwanese were HCV-positive [33,34], the undiagnosed HCV infection rate of the HCV-uninfected cohort might be negligible. Third, the case numbers of patients who developed esophageal cancer were low in the TNHIRD cohort, which might lead to some statistical biases. In particular, the difference between HCV-untreated and HCV-uninfected cohorts could be due to random variation. Fourth, the precise mechanism of the increased risk of esophageal cancer in HCV-infected patients was undetermined, and some documented risks for esophageal cancer, such as alcohol, smoking, betel nut chewing [4], MetS [6,29], hypertension [35], and obesity [7] cannot be identified from the TNHIRD. Fifth, the ICD-9 CM code cannot differentiate ESCC and EAC. The trend of specific histology of esophageal cancer in HCV-infected patients thus cannot be identified. Lastly, as mentioned above, what we found in the current study that adopted interferon-based therapy demands further verification by using DAAs as anti-HCV therapy. Future prospective studies in other independent cohorts with a large number of esophageal cancer cases, definite HCV-uninfected diagnosis confirmed by negative HCV serological tests, identifiable SVR following DAAs with comprehensive risk and histology surveys, and sophisticated molecular investigations are required to elucidate the fundamental mechanisms underlying the findings described here.

Taken together, both HCV positivity and male sex were associated with the development of esophageal cancer. However, whether HCV infection is the true culprit or only a bystander for developing esophageal cancer remains to be further investigated. Interferon-based anti-HCV therapy might attenuate the risk of esophageal cancer development and decrease the mortality of HCV-infected patients.

## Figures and Tables

**Table 1:** Baseline characteristics of the 3 HCV cohorts of TNHIRD.

	(1)	(2)	(3)	*p*-Values		
	HCV-Treated	HCV-Untreated	HCV-Uninfected	(1),(2)	(1),(3)	(2),(3)
*n*	9047	9047	9047			
Gender						
Female, *n*, (%)	4182 (46.23)	4182 (46.23)	4182 (46.23)	1	1	1
Age range (years), *n*, (%)						
20–39	1569 (17.34)	1569 (17.34)	1569 (17.34)	1	1	1
40–49	2515 (27.80)	2515 (27.80)	2515 (27.80)			
50–59	3260 (36.03)	3260 (36.03)	3260 (36.03)			
≥60	1703 (18.82)	1703 (18.82)	1703 (18.82)			
Area, *n*, (%)						
city	2205 (24.37)	2205 (24.37)	2205 (24.37)	1	1	1
township	2785 (30.78)	2785 (30.78)	2785 (30.78)			
rural area	4057 (44.84)	4057 (44.84)	4057 (44.84)			
CCI score, *n*, (%)						
0	4292 (47.44)	4292 (47.44)	4292 (47.44)	1	1	1
1	3029 (33.48)	3029 (33.48)	3029 (33.48)			
≥2	1726 (19.08)	1726 (19.08)	1726 (19.08)			
Index year, *n*, (%)						
2003–2006	4400 (48.63)	4400 (48.63)	4400 (48.63)	1	1	1
2007–2009	2805 (31.00)	2805 (31.00)	2805 (31.00)			
2010–2012	1842 (20.36)	1842 (20.36)	1842 (20.36)			
Baseline factor, *n*, (%)						
Liver cirrhosis	969 (10.71)	546 (6.04)	6 (0.07)	<0.0001	<0.0001	<0.0001
COPD	1050 (11.61)	1017 (11.24)	892 (9.86)	0.4406	0.0001	0.0025
ESRD	61 (0.67)	253(2.80)	23 (0.25)	<0.0001	<0.0001	<0.0001
DM	1702 (18.81)	2051 (22.67)	1677 (18.54)	<0.0001	0.6334	<0.0001
Hypertension	2668 (29.49)	3154 (34.86)	2498 (27.61)	<0.0001	0.0051	<0.0001
Dyslipidemia	1107 (12.24)	1781 (19.69)	1686 (18.64)	<0.0001	<0.0001	0.0727
Cardiovascular events	234 (2.59)	360 (3.98)	255 (2.82)	<0.0001	0.3357	<0.0001
Stroke	298 (3.29)	441 (4.87)	466 (5.15)	<0.0001	<0.0001	0.3944

(1): HCV-treated cohort; (2): HCV-untreated cohort; (3): HCV-untreated cohort; HCV: hepatitis C virus; TNHIRD: Taiwan National Health Insurance Research Database; CCI: Charlson Comorbidity Index; COPD: Chronic obstructive pulmonary disease; ESRD: end stage renal disease; DM: diabetes.

**Table 2:** Comparison of the cumulative incidences of esophageal cancers among (1) HCV-treated, (2) HCV-untreated, and (3) HCV-uninfected cohorts.

	HCV-Treated	HCV-Untreated	HCV-Uninfected	*p*-Value
	*n* = 9047	*n* = 9047	*n* = 9047	
Follow-up years, mean ± SD	2.76 ± 1.75	2.97 ± 1.83	2.96 ± 1.81	
Event number, *n* (%)	1 (0.01)	86 (0.56)	40 (0.26)	
Competing mortality, *n* (%)	209 (2.31)	762 (4.95)	272 (1.77)	
CI, % (95% CI)	0.019 (0.002–0.109)	0.174 (0.068–0.395)	0.035 (0.007–0.133)	0.0292

HCV: hepatitis C virus; SD: standard deviation; CI: cumulative incidence; 95% CI: 95% confidence interval of cumulative incidence 0.007–0.133.

## Data Availability

The datasets generated during and/or analysed during the current study are available from the corresponding author on reasonable request.

## Supplementary Materials

The following are available online at https://www.mdpi.com/article/10.3390/jcm10112395/s1, Figure S1: Matching process of the 3 TNHIRD cohorts, including HCV-treated, HCV-untreated, and HCV-uninfected cohorts, Figure S2: Forest plot of factors associated with esophageal cancers in the 3 TNHIRD cohorts, including HCV-treated, HCV-untreated, and HCV-uninfected cohorts. esoph.: esophageal. Table S1. Baseline characteristics of the 2 HCV cohorts of TNHIRD.

## References

[1] Bray F., Me J.F., Soerjomataram I., Siegel R.L., Torre L.A., Jemal A. (2018). Global cancer statistics 2018: GLOBOCAN estimates of incidence and mortality worldwide for 36 cancers in 185 countries. CA Cancer J. Clin..

[2] Siegel R.L., Mph K.D.M., Jemal A. (2018). Cancer statistics, 2018. CA Cancer J. Clin..

[3] Testa U., Castelli G., Pelosi E. (2017). Esophageal Cancer: Genomic and Molecular Characterization, Stem Cell Compartment and Clonal Evolution. Medicines.

[4] Lee C.-H., Lee J.-M., Wu D.-C., Hsu H.-K., Kao E.-L., Huang H.-L., Wang T.-N., Huang M.-C., Wu M.-T. (2004). Independent and combined effects of alcohol intake, tobacco smoking and betel quid chewing on the risk of esophageal cancer in Taiwan. Int. J. Cancer.

[5] Huang F.-L., Yu S.-J. (2018). Esophageal cancer: Risk factors, genetic association, and treatment. Asian J. Surg..

[6] Zhang J., Wu H., Wang R. (2021). Metabolic syndrome and esophageal cancer risk: A systematic review and meta-analysis. Diabetol. Metab. Syndr..

[7] Chang M.-L., Yang Z., Yang S.-S. (2020). Roles of Adipokines in Digestive Diseases: Markers of Inflammation, Metabolic Alteration and Disease Progression. Int. J. Mol. Sci..

[8] Chang M.-L. (2016). Metabolic alterations and hepatitis C: From bench to bedside. World J. Gastroenterol..

[9] Cheng Y.T., Cheng J.S., Lin C.H., Chen T.-H., Lee K.-C., Chang M.-L. (2020). Rheumatoid factor and immunoglobulin M mark hepatitis C-associated mixed cryo-globulinaemia: An 8-year prospective study. Clin. Microbiol. Infect..

[10] Chang M.-L., Chen W.-T., Hu J.-H., Chen S.-C., Gu P.-W., Chien R.-N. (2020). Altering retinol binding protein 4 levels in hepatitis C: Inflammation and steatosis matter. Virulence.

[11] Chang M.-L., Lin Y.-S., Hsu C.-L., Chien R.-N., Fann C.S. (2021). Accelerated cardiovascular risk after viral clearance in hepatitis C patients with the NAMPT-rs61330082 TT genotype: An 8-year prospective cohort study. Virulence.

[12] Negro F., Forton D., Craxì A., Sulkowski M.S., Feld J.J., Manns M.P. (2015). Extrahepatic Morbidity and Mortality of Chronic Hepatitis C. Gastroenterology.

[13] Allison R.D., Tong X., Moorman A.C., Ly K.N., Rupp L., Xu F., Gordon S.C., Holmberg S.D. (2015). Increased incidence of cancer and cancer-related mortality among persons with chronic hepatitis C infection, 2006–2010. J. Hepatol..

[14] Mahale P., Sturgis E.M., Tweardy D.J., Ariza-Heredia E.J., Torres H.A. (2016). Association Between Hepatitis C Virus and Head and Neck Cancers. J. Natl. Cancer Inst..

[15] Lee M.-H., Yang H.-I., Lu S.-N., Jen C.-L., You S.-L., Wang L.-Y., Wang C.-H., Chen W.J., Chen C.-J., Reveal-HCV Study Group (2012). Chronic Hepatitis C Virus Infection Increases Mortality from Hepatic and Extrahepatic Diseases: A Community-Based Long-Term Prospective Study. J. Infect. Dis..

[16] Chen C.-W., Cheng J.-S., Chen T.-D., Le P.-H., Ku H.-P., Chang M.-L. (2019). The irreversible HCV-associated risk of gastric cancer following interferon-based therapy: A joint study of hospital-based cases and nationwide population-based cohorts. Ther. Adv. Gastroenterol..

[17] Fiorino S., Bacchi-Reggiani L., De Biase D., Fornelli A., Masetti M., Tura A., Grizzi F., Zanello M., Mastrangelo L., Lombardi R. (2015). Possible association between hepatitis C virus and malignancies different from hepatocellular carcinoma: A systematic review. World J. Gastroenterol..

[18] Vermehren J., Park J., Jacobson I.M., Zeuzem S. (2018). Challenges and perspectives of direct antivirals for the treatment of hepatitis C virus infection. J. Hepatol..

[19] Toyoda H., Kumada T., Tada T., Kiriyama S., Tanikawa M., Hisanaga Y., Kanamori A., Kitabatake S., Ito T. (2015). Risk factors of hepatocellular carcinoma development in non-cirrhotic patients with sustained virologic response for chronic hepatitis C virus infection. J. Gastroenterol. Hepatol..

[20] Kalaitzakis E., Gunnarsdottir S.A., Josefsson A., Björnsson E. (2011). Increased Risk for Malignant Neoplasms Among Patients with Cirrhosis. Clin. Gastroenterol. Hepatol..

[21] Hu J.-H., Chen M.-Y., Yeh C.-T., Lin S.-H., Lin M.-S., Huang T.-J., Chang M.-L. (2016). Sexual Dimorphic Metabolic Alterations in Hepatitis C Virus-infected Patients: A Community-Based Study in a Hepatitis B/Hepatitis C Virus Hyperendemic Area. Medicine.

[22] Deyo R.A., Cherkin D.C., Ciol M.A. (1992). Adapting a clinical comorbidity index for use with ICD-9-CM administrative databases. J. Clin. Epidemiol..

[23] Gray R.J. (1988). A Class of K-Sample Tests for Comparing the Cumulative Incidence of a Competing Risk. Ann. Stat..

[24] Fine J.P., Gray R.J. (1999). A Proportional Hazards Model for the Subdistribution of a Competing Risk. J. Am. Stat. Assoc..

[25] https://www.nhi.gov.tw/Content_List.aspx?n=A4EFF6CD1C4891CA&topn=3FC7D09599D25979.

[26] Lu C.-L., Lang H.-C., Luo J.-C., Liu C.-C., Lin H.-C., Chang F.-Y., Lee S.-D. (2009). Increasing trend of the incidence of esophageal squamous cell carcinoma, but not adenocarcinoma, in Taiwan. Cancer Causes Control.

[27] Gunjal S., Pateel D.G.S., Yang Y.-H., Doss J.G., Bilal S., Maling T.H., Mehrotra R., Cheong S.C., Zain R.B.M. (2020). An Overview on Betel Quid and Areca Nut Practice and Control in Selected Asian and South East Asian Countries. Subst. Use Misuse.

[28] Lin C.H., Lin C.C., Liu C.S. (2011). Betel nut chewing as a risk factor for hepatitis C infection in Taiwan—A community-based study. Ann. Saudi Med..

[29] Peng F., Hu D., Lin X., Chen G., Liang B., Zhang H., Dong X., Lin J., Zheng X., Niu W. (2017). Analysis of Preoperative Metabolic Risk Factors Affecting the Prognosis of Patients with Esophageal Squamous Cell Carcinoma: The Fujian Prospective Investigation of Cancer (FIESTA) Study. EBioMedicine.

[30] Dix O., Thakur M., Genova A. (2020). Increased Risk of Esophageal Cancers Among Men in Taiwan. Cureus.

[31] Yu M.-L., Dai C.-Y., Huang J.-F., Hou N.-J., Lee L.-P., Hsieh M.-Y., Chiu C.-F., Lin Z.-Y., Chen S.-C., Wang L.-Y. (2007). A randomised study of peginterferon and ribavirin for 16 versus 24 weeks in patients with genotype 2 chronic hepatitis C. Gut.

[32] Yu M.-L., Huang C.-F., Huang J.-F., Chang N.-C., Yang J.-F., Lin Z.-Y., Chen S.-C., Hsieh M.-Y., Wang L.-Y., Chang W.-Y. (2010). Role of interleukin-28B polymorphisms in the treatment of hepatitis C virus genotype 2 infection in Asian patients. Hepatology.

[33] Cheng Y.L., Wang Y.C., Lan K.H., Huo T.-L., Huang Y.-H., Su C.-W., Lin H.-C., Lee F.-Y., Wu J.-C., Lee S.-D. (2015). Anti-hepatitis C virus seropositivity is not associated with metabolic syndrome irrespective of age, gender and fibrosis. Ann. Hepatol..

[34] Polaris Observatory HCV Collaborators (2017). Global prevalence and genotype distribution of hepatitis C virus infection in 2015: A modelling study. Lancet Gastroenterol. Hepatol..

[35] Seo J.H., Kim Y.D., Park C.S., Han K.-D., Joo Y.-H. (2020). Hypertension is associated with oral, laryngeal, and esophageal cancer: A nationwide population-based study. Sci. Rep..

