# Supplementary material for: Association between Hepatitis C Virus Infection and Esophageal Cancer: An Asian Nationwide Population-Based Cohort Study
Source: J Clin Med. 2021 May 28;10(11):2395. doi: 10.3390/jcm10112395 (PMC8198559; doi:10.3390/jcm10112395)
Supplement: Supplementary file 1 [file jcm-10-02395-s001.zip › jcm-1190816-supplementary.pdf]

Supplementary Materials

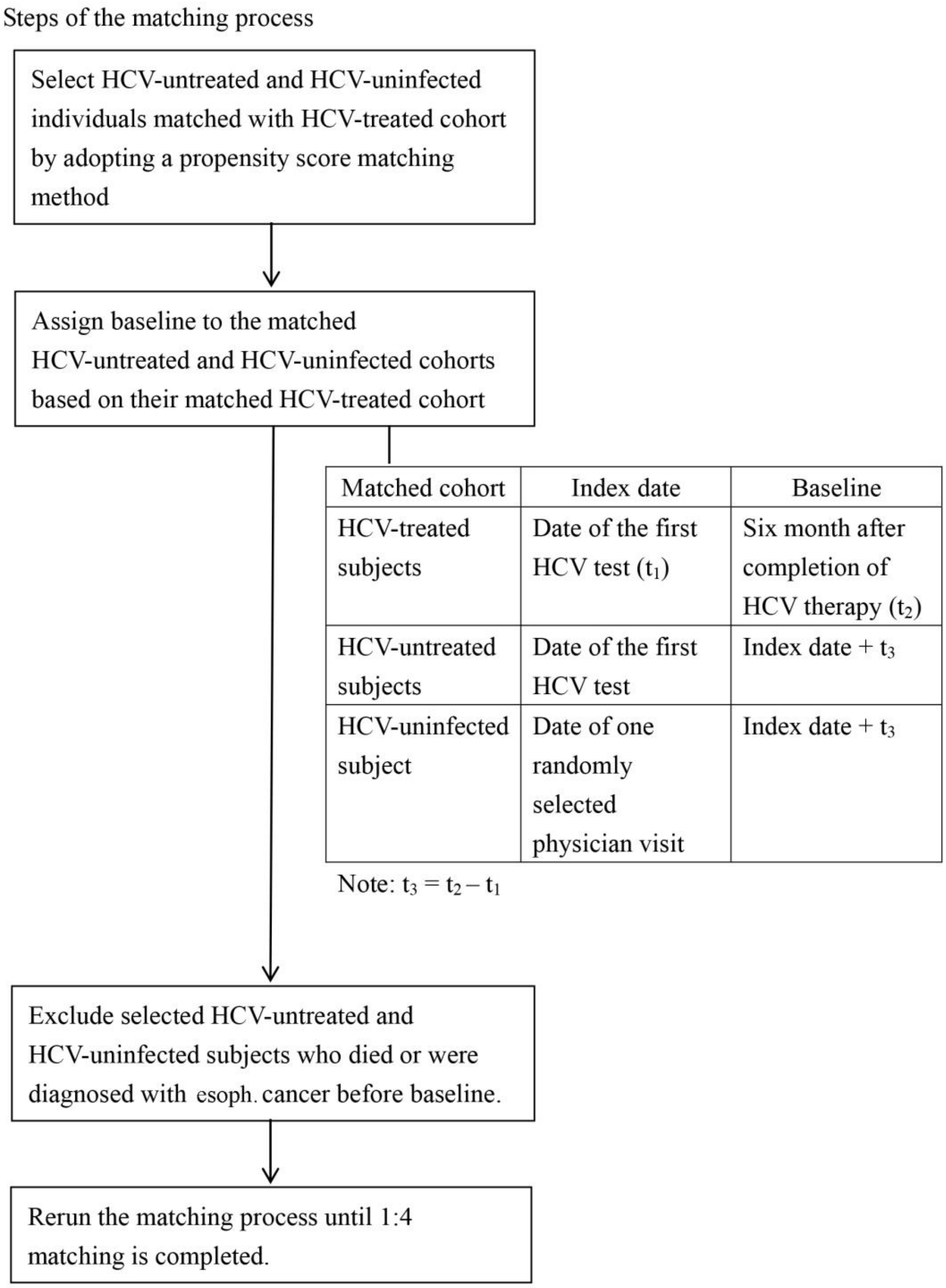

Matched cohort	Index date	Baseline
HCV-treated subjects	Date of the first HCV test (t_1)	Six month after completion of HCV therapy (t_2)
HCV-untreated subjects	Date of the first HCV test	Index date + t_3
HCV-uninfected subject	Date of one randomly selected physician visit	Index date + t_3

Figure S1. Matching process of the 3 TNHIRD cohorts, including HCV-treated, HCV-untreated, and HCV-uninfected cohorts.

(1,2,3) N=27,141

	Esophagus			
	**HR**	**95% LCL**	**95% HCL**	**p-value**
**HCV (ref=untreated)**				
**treated**	0.128	0.016	1.042	0.0547
**uninfected**	0.268	0.057	1.257	0.095
**Gender (ref=Female)**				
**Male**	8.894	1.194	66.227	0.0329
**Age (ref<49)**				
**≥49**	1.561	0.419	5.817	0.5069
**Area (ref=city)**				
**township**	0.514	0.084	3.13	0.4704
**rural area**	1.008	0.263	3.865	0.9907
**CCI score (ref=0)**				
**1**	1.031	0.248	4.29	0.9661
**≥2**	0.880	0.169	4.579	0.8791
**index_yr (ref=2003-2006)**				
**2007-2009**	1.805	0.517	6.304	0.3545
**2010-2012**	0.806	0.091	7.176	0.8468
**Baseline factor (ref=N)**				
**Liver cirrhosis**	1.558	0.201	12.056	0.671
**COPD**	0.783	0.104	5.905	0.8121
**ESRD**	0.000	0	0	
**DM**	1.439	0.43	4.814	0.5546
**Hypertension**	1.208	0.259	5.639	0.8098
**Dyslipidemia**	0.410	0.057	2.977	0.3783
**Cardiovascular events***	0.000	0	0	
**Stroke**	1.767	0.165	18.953	0.6381

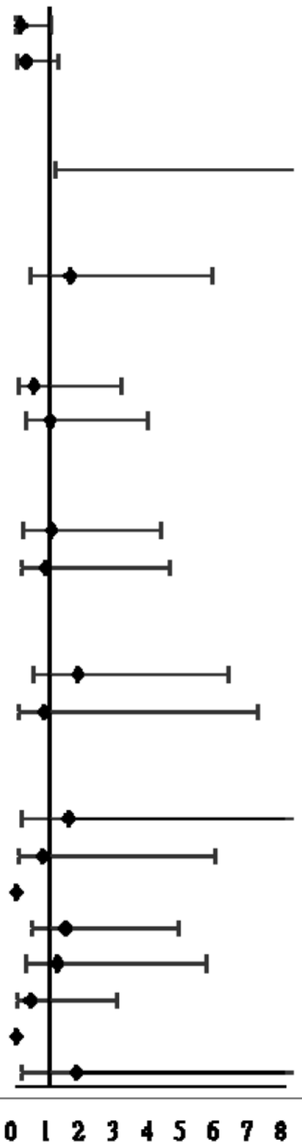

Figure S2. Forest plot of factors associated with esophageal cancers in the 3 TNHIRD cohorts, including HCV-treated, HCV-untreated, and HCV-uninfected cohorts. esoph.: esophageal.
